# Ultrasonic-assisted extraction (UAE) of Javanese turmeric rhizomes using natural deep eutectic solvents (NADES): Screening, optimization, and *in vitro* cytotoxicity evaluation

**DOI:** 10.1016/j.ultsonch.2025.107271

**Published:** 2025-02-12

**Authors:** Donna Maretta Ariestanti, Abdul Mun’im, Pietradewi Hartrianti, Basmah Nadia, Erika Chriscensia, Shereen Angelina Rattu, Redhalfi Fadhila, Anastacia Harianto, Adelina Simamora, Delly Ramadon, Richard Johari James, Fadlina Chany Saputri, Mitsuyasu Kato, Meidi Utami Puteri

**Affiliations:** a Faculty of Pharmacy, Universitas Indonesia, Kampus UI, Depok, West Java 16424, Indonesia; b National Metabolomics Collaborative Research Center, Faculty of Pharmacy, Universitas Indonesia, Kampus UI, Depok, West Java 16424, Indonesia; c Department of Pharmacy, School of Life Sciences, Indonesia International Institute for Life Sciences (I3L), Jakarta, Indonesia; d Department of Biomedicine, School of Life Sciences, Indonesia International Institute for Life Sciences (I3L), Jakarta, Indonesia; e Integrative Pharmacogenomics Institute, Universiti Teknologi MARA Cawangan Selangor, Puncak Alam, Selangor, Malaysia; f Faculty of Pharmacy, Universiti Teknologi MARA Cawangan Selangor, Puncak Alam, Selangor, Malaysia; g Department of Experimental Pathology, Graduate School of Comprehensive Human Sciences and Faculty of Medicine, University of Tsukuba, 1-1-1 Tennodai, Tsukuba, Ibaraki 305-8575, Japan

**Keywords:** C. xanthorrhiza, Javanese turmeric, In vitro cytotoxicity, NADES, Response surface methodology, Ultrasound-assisted extraction (UAE)

## Abstract

Javanese turmeric (*Curcuma xanthorrhiza* Roxb.) is known for its diverse pharmacological activities due to its rich phytoconstituents, including curcuminoids and xanthorrhizol. Typically, these compounds are extracted using organic solvents, which pose health and environmental risks. Therefore, safer and more environmentally friendly green extraction methods are being developed. This study investigated the effect of ultrasound-assisted extraction (UAE) combined with natural deep eutectic solvents (NADES) based on choline chloride and organic acids (lactic, malic, and citric acid) to find the best combination for extracting curcuminoids and xanthorrhizol from Javanese turmeric. Results showed that UAE using choline chloride and malic acid (1:1) (ChCl-MA) yielded the best results. The Box–Behnken Design optimized water addition, solvent-to-powder ratio, and extraction time, with optimal conditions being 25 % water addition, a 20 mL/g ratio, and a 15-minute extraction time. This method yielded 4.58 mg/g of curcuminoids and 12.93 mg/g of xanthorrhizol. Furthermore, the ChCl-MA NADES with UAE extraction showed more cytoselective activity towards the HeLa cancer cell line compared to the non-cancer HaCaT cell line. In contrast, traditional ethanol extraction was non-selective, as indicated by similar cell viability reductions in both HeLa and HaCaT cells at 6.25 ppm. Collectively, this study is the first to report the optimal NADES combination with UAE, based on salts and organic acids, for the extraction of Javanese turmeric rhizomes with selective cytotoxic effects against cancer cells. These findings may contribute to the development of novel, naturally derived anticancer agents using green extraction techniques.

## 1 Introduction

Javanese turmeric (*Curcuma xanthorrhiza* Roxb.) also known as Javanese ginger, or Temulawak in Indonesia, is a plant native to Indonesia. The rhizomes of Javanese turmeric has been used as an ingredient in herbal medicines, supplements, and Indonesian herbal remedies. Additionally, it is cultivated in Malaysia, Sri Lanka, Thailand, and the Philippines [1]. The major components of Javanese turmeric rhizomes are curcuminoids and xanthorrhizol [2]. It has been reported that curcuminoids have anti-inflammatory, antibacterial, anti-tumor, and anti-diabetic properties [3]. Xanthorrizol has been shown in studies to have antioxidant, antibacterial, anti-inflammatory, antihyperglycemic, antihypertensive, antiplatelet, nephroprotective, and hepatoprotective characteristics, as well as estrogenic and antiestrogenic properties [4].

The phytoconstituents in Javanese turmeric rhizomes are typically extracted using organic solvents [5]. However, organic solvents are considered unsafe for the body and harmful to the environment when used in large quantities [6]. Therefore, alternative extraction methods have been developed to more efficiently extract curcuminoid and xanthorrhizol from Javanese turmeric rhizomes. Natural deep eutectic solvents (NADES) provide an environmentally friendly alternative for extracting plant bioactive chemicals. NADES are created by combining halide salts (hydrogen bond acceptors) with components acting as hydrogen bond donors. They offer high extraction capabilities, are easy to prepare, non-toxic, non-volatile, and economical. Additionally, NADES can enhance the solubility and bioactivity of water-insoluble compounds like curcumin and xanthorrhizol [7], [8]. It is reported that the best NADES combinations, consisting of organic acids, sugars, or amino acids, can effectively dissolve and extract numerous compounds [8]. Besides the choice of solvent, the extraction method also impacts the extraction of a plant's phytoconstituents. Ultrasound-assisted extraction (UAE) has been shown to be more efficient than conventional methods. Ultrasonic waves in UAE enhance the penetration of NADES into the plant cell matrix, yielding higher extraction levels. Additionally, NADES-UAE extraction requires less time and solvent [9]. This aligns with the concept of green extraction, which minimizes environmental impact by reducing solvent consumption, extraction time, energy, and costs, and using non-toxic solvents [10].

Considering the notable pharmacological potency of curcuminoid and xanthorrhizol in Javanese turmeric rhizomes and their potential as nutraceuticals, we propose adopting NADES with UAE for the selective extraction of these compounds. NADES-UAE may provide a viable, eco-friendly alternative that could improve the efficiency of extracting curcuminoid and xanthorrhizol from Javanese turmeric. A previous study by Rachmaniah et al. [11] found that the most effective NADES combination for extracting curcuminoids from Javanese turmeric was a mixture of choline chloride and malic acid, which yielded the highest extraction efficiency. However, the UAE technique has not been applied in that study and no further bioactivity assay was evaluated to compare the developed and conventional techniques.

Therefore, in this study, we will screen various combinations of choline chloride and organic acids to identify the best mixture for extracting curcuminoids and xanthorrhizol from Javanese turmeric rhizomes with UAE. We will then evaluate the effects of water addition, solvent-to-powder ratio, and UAE time. Furthermore, we will compare the NADES-UAE method with the conventional ethanol (96 %) maceration technique by assessing the cytotoxic effects on non-cancer and cancer cells to evaluate the bioactivity of the extracts produced.

## 2 Materials and methods

### 2.1 Plant and chemicals

Javanese turmeric rhizomes powder was purchased from Ballitro, Indonesia. Chemicals used in this study were choline chloride (Xi’an Rongsheng, China), citric acid (Pudak Scientific, Indonesia), malic acid (Sigma-Aldrich, USA), lactic acid (Sigma-Aldrich, USA), Curcuminoid standard (≥95 %, Xi’an, China), xanthorrhizol standart (ITB, Indonesia), ethanol, dichloromethane pro analysis, chloroform pro analysis (Merck, Germany), methanol, aquademin (Brataco, Indonesia). HeLa and HaCaT cells were obtained from Indonesia International Institute for Life Science (i3L) cell collection. Dulbecco’s Modified Eagle’s Medium (DMEM), fetal bovine serum (FBS), penicillin–streptomycin (pen-strep) and trypsin-EDTA 0.25 % were purchased from Gibco (Gibco, USA).

### 2.2 Preparation and characterization of simplicia from Javanese turmeric

Characterization of dried Javanese turmeric rhizome simplicia was done to verify the identity of the plant. The analysis was performed by observing the constituent fragments under a light microscope.

### 2.3 NADES extraction using ultrasound-assisted extraction (UAE) process of Javanese turmeric rhizomes

The preparation of natural deep eutectic solvents (NADES) involved mixing hydrogen bond acceptors (e.g., choline chloride) with hydrogen bond donors (e.g., organic acids) under controlled heating and stirring conditions. Three NADES formulations were prepared using choline chloride and organic acids (malic acid, lactic acid, and citric acid), as detailed in Table 1. The mixtures were heated at 70 °C for 30–90 min until a eutectic solution formed. Subsequently, aquademin was added and the mixture was further heated until a clear, homogeneous liquid was obtained. The composition and molar ratios used were adapted from previous studies that demonstrated the effective extraction of curcuminoids using NADES [9], [11]. For screening the most efficient NADES, extraction parameters were standardized across all three formulations. These included the addition of 25 % water, an extraction time of 20 min, and a solid-to-solvent ratio of 1:15. Javanese turmeric extraction was performed using an ultrasonic-assisted extraction (UAE) bath type (60 kHz, 170 W; Krisbow, China). Briefly, 1 g of Javanese turmeric powder was mixed with the prepared NADES containing 20–25 % water at a solvent-to-powder ratio of 15–20 mL/g. The mixtures were subjected to UAE under the conditions specified in the experimental design (Table 2). After extraction, the samples were centrifuged for 17 min and filtered to obtain the final extract.

**Table 1:** Molar ratio of NADES solvents.

**NADES components**	**NADES codes**	**Molar ratio**
Choline chloride − Malic acid	ChCl-MA	1:1
Choline chloride − Lactic acid	ChCl-LA	1:1
Choline chloride − Citric acid	ChCl-CA	1:1

**Table 2:** Optimization extraction conditions.

**Run**	**Water added (%)**	**Time (min)**	**Solvent to powder ratio (mL/g)**
1	25	15	10
2	25	20	15
3	25	25	10
4	25	20	15
5	25	15	20
6	20	20	20
7	20	25	15
8	30	20	10
9	25	20	15
10	30	25	15
11	25	25	20
12	20	15	15
13	25	20	15
14	20	20	10
15	25	20	15
16	30	15	15
17	30	20	20

### 2.4 Extraction optimization using response surface methodology

The response surface methodology (RSM) using a Box–Behnken Design (BBD) was implemented with Design Expert software version 13 (Stat-Ease, Inc., Minneapolis, MN, USA) to analyze the interactive effects of three factors on the yield of curcuminoids and xanthorrhizol. These factors were water addition, solid-to-liquid ratio, and ultrasonic extraction time. A total of 17 experiments were conducted, as detailed in Table 2. To generate an experimental model correlating the responses with the three independent variables, a second-order polynomial equation was employed as described previously. The relationship between these variables and the responses was examined using analysis of variance (ANOVA) at a significance level of p < 0.05, available in Design-Expert version 13.

### 2.5 Maceration extraction of Javanese turmeric

Extraction using a conventional method was done to compare the extraction result with NADES extraction. The Javanese turmeric rhizomes powder was extracted by maceration method with ethanol 96 % as the solvent (1:10) for 24 h. The extract was filtered using 0.45 μm filter paper and concentrated through a rotary evaporator. Percent yield was calculated with the following formula:

$$Yield\;\left(\%\right)\;=\frac{\mathrm{weightoftheextractobtained}}{\mathrm{weightofthesimpliciaused}}x100\%$$

### 2.6 Cell culture

HeLa and HaCaT cells were cultured in DMEM containing 10 % FBS and 1 % Pen-Strep. The cells were incubated in a humidified atmosphere at 37℃ with 5 % CO_2_. Cells were split using 0.25 % Trypsin-EDTA at 80–90 % confluency.

### 2.7 In-vitro cytotoxicity assay

Cells were seeded into flat-bottomed 96-well plates at a density of 5,000 cells/well. After 24 h of incubation, the medium was removed and replaced with media containing different concentrations of optimized NADES extract (ChCl-MA) and ethanol extracts (SPL) treatments. 5-fluorouracil was used as positive control. The cells were incubated at 37℃ for 48 h.After treatment, cells were washed twice with DMEM. Briefly, 100 μL of complete DMEM and 20 μL of MTS reagent were added to each well and incubated at 37℃ with 5 % CO_2_ for 3 h. Following incubation, the absorbance was measured at 490 nm on a plate reader (The Infinite®M200 NanoQuant, TECAN, Switzerland). Cell viability was measured in percentage according to the equation:

$$\mathrm{cellviability}\left(\%\right)=\frac{\mathrm{Abs}(sample)-Abs(blank)}{\mathrm{Abs}(negativecontrol)-Abs(blank)}\times 100\%$$

### 2.8 Statistical analysis

The statistical significance of the antiproliferative studies was carried out using GraphPad Prism 9.3.1. The normality of data distribution was analyzed by the Shapiro-Wilk test. The data were normally distributed and thus were analyzed using one-way ANOVA, accompanied with Dunnet’s post-hoc testing, where a p-value of less than 0.05 was considered statistically significant.

## 3 Results

### 3.1 NADES extraction of Javanese turmeric rhizomes using UAE Process: Preparation and screening

#### 3.1.1 Macroscopic and microscopic observation of dried Javanese turmeric rhizomes

**Fig. 1:**
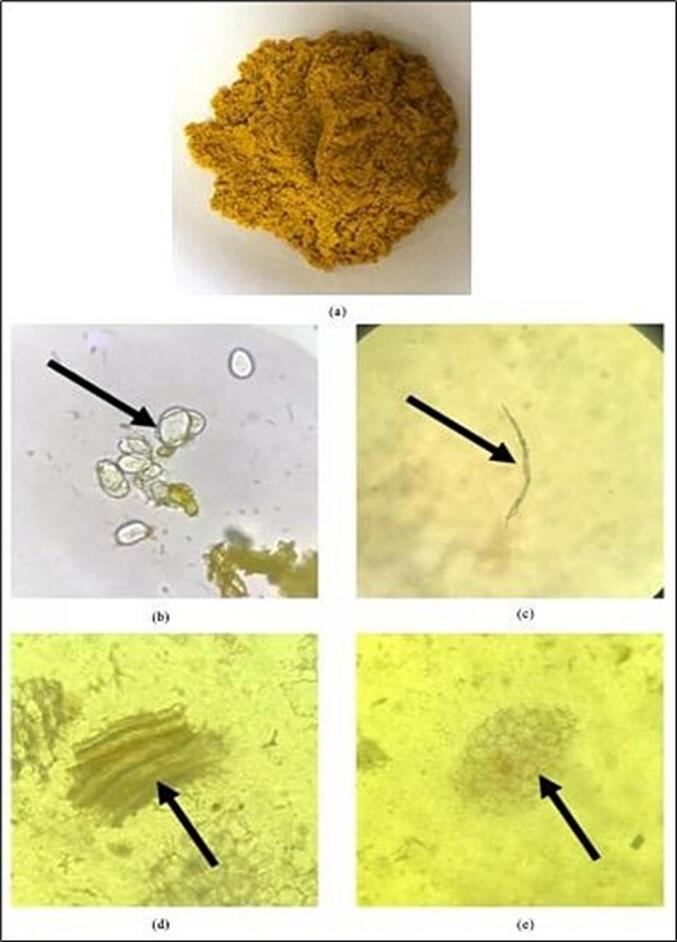
Javanese turmeric rhizome powder. (a) Macroscopic observation; (b) Morphology of Javanese turmeric simplica under a light microscope of amylum; (c) sclerenchyma fibers; (d) transport tissue; (e) cambium.

The sample used in this study was Javanese turmeric rhizome. Sample was further dried, grounded, and sieved to a size of 60 mesh. The particle size reduction was done to increase the contact surface area between the cell matrix and the solvent, which can enhance diffusivity and extraction efficiency [12]. The dried and powdered Javanese turmeric rhizome are shown in Fig. 1a. Next, a microscopic observation of Javanese turmeric powder was done to identify and verify the fragments in our sample. At 40x magnification, characteristic fragments were observed that align with the description in the Indonesian Herbal Pharmacopoeia II [13]. The identified fragments included starch grains (amylum), sclerenchyma fibers, transport tissues, and polygonal cork cambium, as shown in Fig. 1b-e. This observation verified our sample as *Curcuma xanthorrhiza* Roxb.

#### 3.1.2 NADES preparation for Javanese turmeric rhizomes

**Fig. 2:**
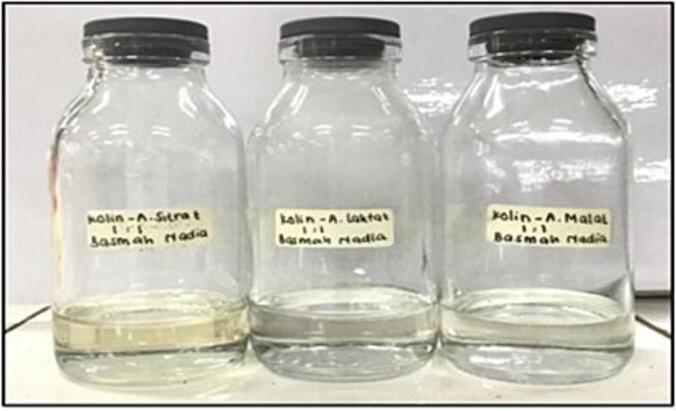
The physical appearance of NADES solvents from left to right: ChCl-CA, ChCl-LA, and ChCl-MA; ChCl-CA: Choline chloride–citric acid, ChCl-LA: Choline chloride–lactic acid, and ChCl-MA: Choline chloride–malic acid.

The NADES solvent used to extract powdered Javanese turmeric rhizome in this study consists of a combination of choline chloride-based NADES and organic acids, as shown in Table 1. The three types of organic acids used are malic acid, lactic acid, and citric acid. In the preparation of NADES solvents, choline chloride acts as the hydrogen bond acceptor, while the organic acids function as hydrogen bond donor. The formation of NADES from choline chloride and organic acids, heated using a hotplate stirrer until a clear eutectic liquid is formed, can be seen in Fig. 2.

#### 3.1.3 Screening of three NADES-UAE extracts of Javanese turmeric rhizome

The screening was conducted on NADES based on choline chloride-organic acid (malic acid, lactic acid, and citric acid) using a ratio of 1:15 (g/mL) between the powder and solvent. One gram of *Javanese turmeric* rhizome powder was weighed and mixed with 15 ml of each NADES solution. The NADES was prepared by adding 25 % water, and the extraction process was performed for 20 min using UAE. These water addition and time conditions were determined after pre-optimization. The combination of choline chloride with these three types of organic acids was selected because it produces NADES with high solubility for curcuminoids [11].

**Fig. 3:**
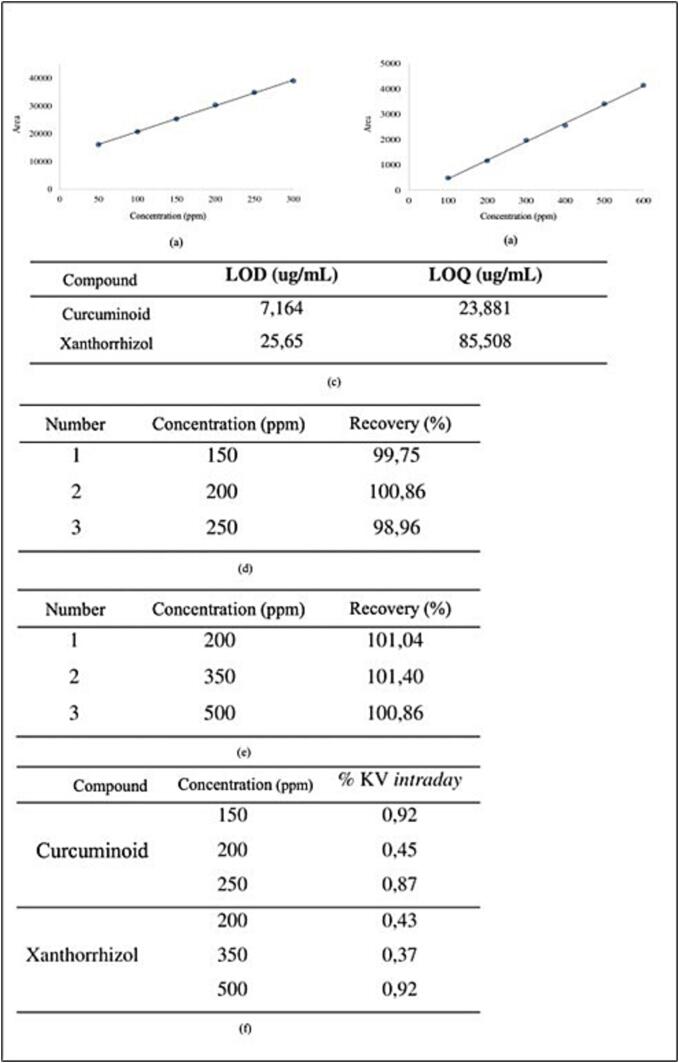
Validation data for curcuminoid and xanthorrizol content using TLC-densitometry.

To quantify the curcuminoid and xanthorrhizol contents in the three NADES-UAE extracts, we employed TLC densitometry for precise measurement of these compounds. A previously validated method was used to ensure accuracy in the analysis. Key validation parameters such as linearity, Limit of Detection (LOD), Limit of Quantification (LOQ), accuracy, and precision were evaluated (Fig. 3). Based on these assessments, the method was confirmed as valid for determining the curcuminoid and xanthorrhizol contents in the extracts.

**Fig. 4:**
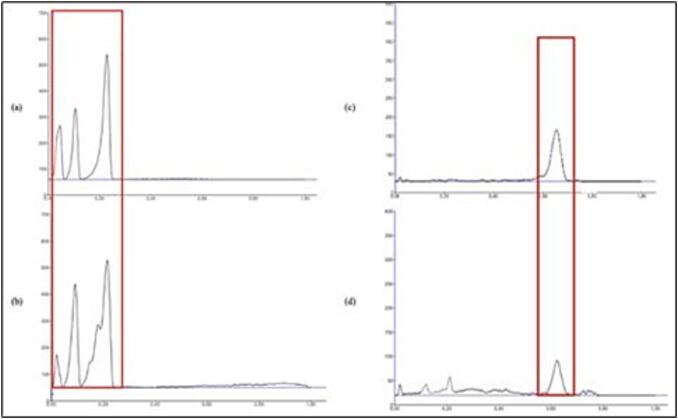
Analysis of Total Curcuminoid and Xanthorrhizol Contents in Extracts Using TLC Densitometry. The analysis was conducted using TLC densitometry with a mobile phase of dichloromethane-chloroform (4:6) and a stationary phase of silica gel 60 F254. The samples analyzed was: (a) Curcuminoid standard at 150 ppm (b) Curcuminoid extract from NADES-UAE (c) Xanthorrhizol standard at 300 ppm (d) Xanthorrhizol extract from NADES-UAE. In the densitometry results, the x-axis represents the wavelength, while the y-axis represents the peak area, indicating the concentration of curcuminoids and xanthorrhizol in the samples.

Next, Analysis was done using CAMAG TLC Scanner 3 at 425 nm wavelength for curcuminoid and 224 nm for xanthorrhizol. Three types of curcuminoid were analyzed; curcumin (CUR), demethoxycurcumin (DMC) and bisdemethoxycurcumin (BDMC). Based on the result, the Rf values of curcuminoid standard for CUR, DMC, and BDMC were 0.25; 0.13; and 0.06, respectively and Rf value for xanthorrhizol standard was 0.68. The samples (one of our NADES-UAE: ChCl-MA extracts) show three peaks at 425 nm wavelength with Rf 0.24; 0.11; and 0.05, and one peak at 224 nm with Rf 0.66 as shown in Fig. 4. This shows that the sample contains three types of curcumin and xanthorrhizol.

**Fig. 5:**
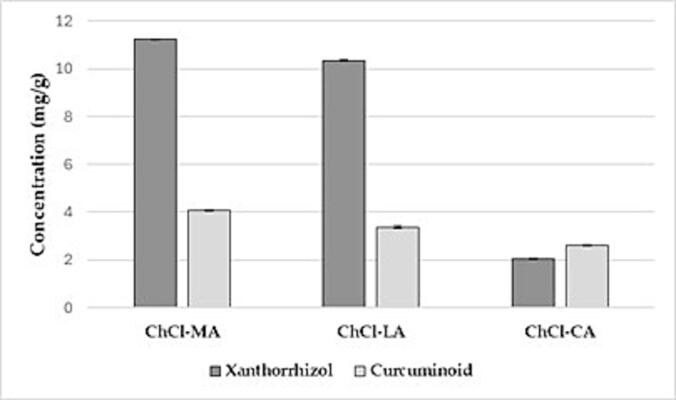
Analysis of Curcuminoid and Xanthorrhizol contents from various NADES-UAE Extracts of Javanese turmeric by TLC-Densitometry (mean ± SD, n = 3).

Based on the analysis, the NADES that produced the highest levels of curcuminoid and xanthorrhizol compounds was the combination of choline chloride with malic acid. The highest concentrations of curcuminoid and xanthorrhizol were 4.08 mg/g and 11.24 mg/g, respectively. The order of compound concentrations from highest to lowest was obtained from the NADES combinations ChCl-MA, ChCl-LA, and ChCl-CA (ChCl-MA > ChCl-LA > ChCl-CA). This indicates that the combination of choline chloride and malic acid is more effective in extracting the total curcuminoid and xanthorrhizol compounds compared to the other combinations. A comparison of the curcuminoid and xanthorrhizol concentrations produced by the three NADES combinations can be seen in Fig. 5. Our study in line with the previous study which also showed that the combination of choline chloride with malic acid is the best combination compare to citric acid.

Our results demonstrate that NADES, particularly the combination of choline chloride and malic acid, significantly enhance the extraction of curcuminoids and xanthorrhizol. This aligns with the growing interest in NADES as eco-friendly, non-toxic, and versatile solvents for bioactive compound extraction. Their unique physicochemical properties, especially their ability to form hydrogen bonds, contribute to the high solubility and extraction efficiency observed in this study. However, challenges such as high viscosity and the need for optimal composition must be addressed to maximize extraction efficiency. By evaluating key parameters—including solvent viscosity, water content, powder-to-solvent ratio, and extraction time—we identified conditions that significantly improve extraction yields. These optimizations not only enhance the recovery of curcuminoids and xanthorrhizol but may also increase their bioactivity, further supporting the potential of NADES as great green extraction solvents.

NADES are naturally occurring eutectic mixtures formed through hydrogen bonding interactions between a hydrogen bond donor and a hydrogen bond acceptor. This interaction lowers the melting point of the mixture compared to that of the individual components [15]. One notable feature of NADES is their intrinsically high viscosity, which can impede the diffusion of solutes and the penetration of solvent into plant matrices, ultimately leading to a decline in extraction efficiency [16], [17]. According to the Stokes-Einstein equation, solute diffusivity is inversely proportional to solvent viscosity, meaning that high-viscosity solvents like NADES hinder molecular movement, thereby decreasing mass transfer efficiency and extraction rates [18], [19]. Furthermore, effective solvent penetration is essential for breaking down plant cell walls and accessing intracellular compounds. However, excessive viscosity impairs penetration, further limiting mass transfer efficiency [20].

Despite these inherent challenges, NADES exhibits robust solvating capabilities and customizable properties, which can enhance solute solubility and partially mitigate the negative effects of reduced diffusivity [21]. Therefore, optimizing a strategy to balance viscosity management while preserving the advantageous characteristics of NADES becomes essential. Research has demonstrated that the introduction of water into NADES can significantly lower viscosity, thereby enhancing mass transfer and extraction efficiency [18], [21].

In this context, the addition of water to NADES emerges as an effective method for viscosity reduction. This incorporation not only improves solvent diffusivity and penetration but also boosts mass transfer and solute solubility, facilitating the efficient extraction of bioactive compounds [22]. Therefore, optimizing viscosity through the inclusion of water is essential for achieving a balance between effective extraction, compound stability, and maximal recovery of bioactives, thereby unlocking the full potential of NADES-based environmentally friendly extraction techniques [21].

In addition to viscosity optimization, the selection of organic acids used in NADES formulations significantly influences hydrogen bonding interactions and extraction efficiency. The acids used in this study for NADES formulations are citric, malic, and lactic acids which contain functional groups capable of donating or accepting electrons, enabling hydrogen bond formation with curcuminoid and xanthorrhizol compounds.

The acids used in this study for NADES formulations are citric, malic, and lactic acids which contain functional groups capable of donating or accepting electrons, enabling hydrogen bond formation with curcuminoid and xanthorrhizol compounds. Citric acid, with multiple carboxyl groups, exhibits a higher capacity for hydrogen bonding than malic acid (which has one hydroxyl group with dicarboxylate) and lactic acid (which has one hydroxyl group with monocarboxylate) [15]. NADES that contain citric acid, therefore, demonstrate a greater potential for forming hydrogen bonds, leading to higher efficiency in extracting curcuminoids compared to NADES with malic or lactic acid [15]. However, while hydrogen bonding is crucial, viscosity also plays a critical role in the extraction process, as high-viscosity NADES can hinder the mass transfer between solvent and sample matrix.

Among the NADES combinations tested, ChCl-CA exhibited the highest viscosity, followed by ChCl-MA and ChCl-LA. This high viscosity can slow down mass transfer, resulting in longer and potentially less efficient extraction. Our findings indicate that the ChCl-MA combination achieved the highest extraction efficiency compared to ChCl-LA and ChCl-CA. This efficiency is likely due to ChCl-MA’s optimal balance between hydrogen bonding capacity and moderate viscosity, which allows for effective interaction with target compounds, leading to higher extraction yields of curcuminoids and xanthorrhizol.

### 3.2 Optimization of NADES-UAE (ChCl-MA) using response surface methodology (RSM) for Javanese turmeric rhizome extraction

Response Surface Methodology (RSM) was done to design the experiment with the significant parameters that affect the optimum extraction response. Analysis was done to the sample extracted with choline chloride-malic acid NADES. Content from the sample was determined as response and analyzed using Design Expert-13 software. From 17 runs, the optimum extraction condition lies at run 5 where it yielded the highest curcuminoid and xanthorrhizol content. The optimum extraction conditions were 25 % water addition, 15 min extraction time, 1:20 (gr/mL) powder to solvent ratio that produced 4.58 mg/g of curcuminoid and 12.93 mg/g of xanthorrhizol (Table 3).

**Table 3:** Acquisition data of Curcuminoid and Xanthorrhizol contents.

**Run**	**Percentage of water added (%)**	**Extraction time (min)**	**Powder to solvent ratio (mL/g)**	**Curcuminoid content** **(mg/g)**	**Xanthorrhizol content** **(mg/g)**
1	25	15	10	2.69	5.37
2	25	20	15	3.98	11.82
3	25	25	10	3.38	5.56
4	25	20	15	4.55	11.30
**5**	**25**	**15**	**20**	**4.58**	**12.93**
6	20	20	20	3.84	11.19
7	20	25	15	3.34	10.10
8	30	20	10	2.72	5.28
9	25	20	15	4.10	11.56
10	30	25	15	4.01	11.99
11	25	25	20	4.51	12.05
12	20	15	15	3.92	9.44
13	25	20	15	4.53	11.48
14	20	20	10	1.64	4.97
15	25	20	15	4.38	10.03
16	30	15	15	4.12	11.49
17	30	20	20	4.31	12.53

Based on the software, the recommended analytical model has a p-value < 0.05, indicating that the model provides a statistically significant response in the ANOVA test. Overall, the model showed a significant response to curcuminoid levels (p = 0.0015) and xanthorrhizol levels (p < 0.0001) (Supplementary Tables 1&2). The p-values for the lack-of-fit test was non-significant (p > 0.05), indicating that the response data for the concentrations fit well with the model [14]. The response analysis model for curcuminoid and xanthorrhizol concentrations used a quadratic model, as recommended by the software.

Significant variables were identified with p-values < 0.05. Variables that had a significant effect on curcuminoid concentration response were A (percentage of water addition), A^2^ (the square of the percentage of water addition), C (powder-to-solvent ratio), and C^2^ (the square of the powder-to-solvent ratio). Based on the model, this equation describes the relationship between the variables and the curcuminoid concentration response:

$$\text{y}\;=-19.9942+1.18247\text{A}\;-0.16014\text{B}\;+1.21554\text{C}\;+0.00459516\text{AB}\;-0.00605359\text{AC}\;-0.00762714\;\text{BC}\;-0.0224631{\text{A}}^{2}+0.00394698{\text{B}}^{2}-0.0247254{\text{C}}^{2}$$

The factors that had significant impact on xanthorrhizol concentration response were A (percentage of water addition), C (powder-to-solvent ratio), and C^2^ (the square of the powder-to solvent ratio). The following equation describing the relationship between the variables and the

xanthorrhizol concentration response:

$$\text{y}\;=-28.894+0.717422\text{A}\;-0.127557\text{B}\;+3.35392\text{C}\;-0.00017852\text{AB}\;+0.00747309\text{AC}\;-0.0089788\text{BC}\;-0.0137746{\text{A}}^{2}+0.00711305{\text{B}}^{2}-0.0892299{\text{C}}^{2}$$

Note:y = Xanthorrhizol concentration response (dependent variable).

A = Percentage of water addition (independent variable).

B = Extraction time (independent variable).

C = Powder-to-solvent ratio (independent variable).

A^2^, B^2^, C^2^ = Square of the respective variables, representing quadratic effects.

AB, AC, BC = Interaction effects between the variables.

According to the analysis, the addition of water to NADES was the first variable that significantly influenced extraction yield. Reducing NADES viscosity by adding water helps increase solvent diffusivity into the sample matrix, improving mass transfer and reducing extraction times [23]. However, excessive water addition can dilute the hydrogen bonding interactions between NADES and the target compounds, potentially reducing extraction efficiency [24], [25]. In our study, 25 % water addition represented an optimal condition for NADES ChCl-MA, yielding the highest levels of curcuminoids and xanthorrhizol compared to 20 % and 30 % water additions. The powder-to-solvent ratio was another variable with a significant impact on the extraction efficiency of curcuminoids and xanthorrhizol using NADES-UAE (p < 0.05). In this study, the highest tested ratio, 1:20 g/mL, was found to be optimal. Increasing the powder-to-solvent ratio generally enhanced compound solubility in the solvent, leading to higher extraction yields. A larger surface area facilitated by a high powder concentration increases solute–solvent interactions, enhancing extraction efficiency [26]. However, excessive solvent relative to the powder can stabilize mass transfer without further yield improvements, making additional solvent use inefficient. Moreover, extraction time did not significantly affect curcuminoid or xanthorrhizol concentrations in this NADES-UAE process, as shown by non-significant p-values (p > 0.05) in ANOVA results. The optimal extraction time was determined to be 15 min. The lack of significant impact might be attributed to the narrow time range tested, which may not fully capture the influence of extraction duration on compound yield.

### 3.3 The comparison of NADES-UAE extraction results with ethanol maceration

In this study, conventional extraction methods were applied to extract Javanese turmeric rhizome powder. The aim was to compare the total curcuminoid content and xanthorrhizol yield between traditional ethanol maceration and green extraction using NADES-UAE. The NADES-UAE ChCl-MA combination was chosen for further experiments based on the results of our screening and optimization process. The maceration process involved 50 g of Javanese turmeric rhizome powder immersed in 500 mL of 96 % ethanol. The first maceration was carried out for 24 h, followed by two additional maceration cycles. The resulting extract was concentrated using a rotary evaporator until a thick extract was obtained, yielding 4.47 g of extract with an overall yield of 8.91 %.

**Fig. 6:**
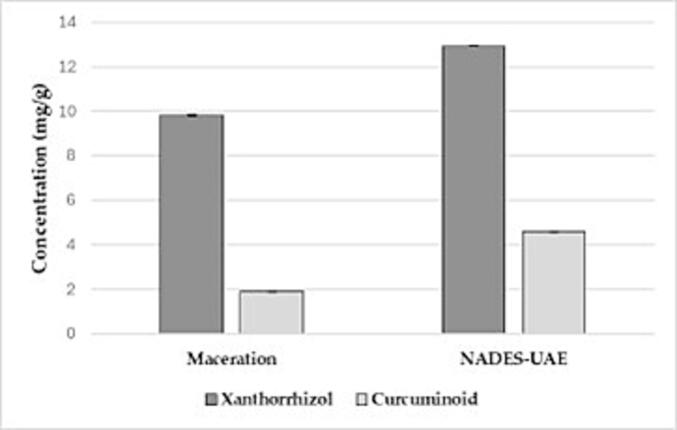
The comparison of yield obtained from the NADES-UAE vs ethanol maceration extraction method (mean ± SD, n = 3).

The comparison of total curcuminoid content and xanthorrhizol yield between the NADES-UAE ChCl-MA and ethanol maceration methods is illustrated in Fig. 6. The conventional maceration process yielded 1.88 mg/g of curcuminoid and 9.80 mg/g of xanthorrhizol. In contrast, extraction using the NADES-UAE ChCl-MA method resulted in significantly higher yields, with 4.58 mg/g of curcuminoid and 12.93 mg/g of xanthorrhizol.

We found the comparatively lower yields of curcuminoids and xanthorrhizol obtained via traditional ethanol maceration, this may be due to the solvent's saturation, which limits its extraction capacity. Differences in solvent volume and powder-to-solvent ratios also play a role in the efficiency of maceration. In contrast, NADES-UAE with ChCl-MA showed higher yields, likely due to the cavitation effects generated by ultrasonic waves. These cavitation bubbles disrupt cell walls, allowing the rapid diffusion of target compounds into the NADES [27]. Consequently, the NADES-UAE ChCl-MA method enhances the extraction of curcuminoids and xanthorrhizol from Javanese turmeric with shorter extraction times and less solvent use than traditional maceration.

### 3.4 Effects of extraction condition on in vitro cytotoxicity of Javanese turmeric extract on HeLa and HaCaT cells

**Fig. 7:**
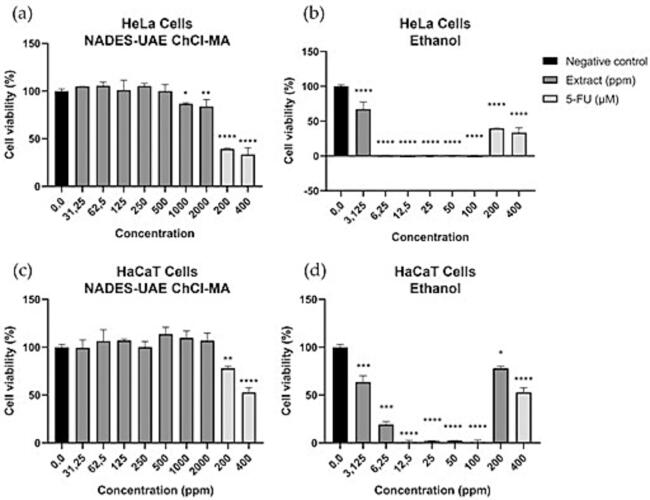
Cell viability (%) of HaCaT and HeLa cells after 48 h of extract treatment. The result was compared to negative controls where the cells were not treated with DES-6, DES-14 or SPL and expressed as mean ± standard deviation of triplicates sample using one-way ANOVA, post-hoc Tukey. * Indicate statistical difference (P < 0.1). ** Indicate statistical significant difference (P < 0.01). *** Indicate statistical significant difference (P < 0.001) **** Indicate statistical significant difference (P < 0.0001).

To further evaluate the differences between NADES-UAE and the conventional extraction method, various concentrations of NADES-UAE ChCl-MA extracts and ethanol extracts, along with the positive control (5-fluorouracil), were administered to HeLa, a cervical cancer cell line, and HaCaT, immortalized human keratinocytes. After 48 h, cell viability was measured, as shown in Fig. 7. Based on the results, the NADES-UAE ChCl-MA extract exhibited selective cytotoxicity toward HeLa cells, significantly reducing viability at 1000 ppm (*p* < 0.05), with a more pronounced effect at 2000 ppm (*p* < 0.0001). In contrast, NADES-UAE ChCl-MA did not affect HaCaT cell viability until 2000 ppm (*p* < 0.01). Meanwhile, ethanol extracts displayed broad cytotoxic activity, significantly decreasing viability in both HeLa and HaCaT cells at the lowest tested concentration, starting from 3.125 ppm to 2000 ppm (*p* < 0.0001).

Our findings highlight the anticancer potential of curcuminoids and xanthorrhizol extracted from Javanese turmeric. Curcuminoids have been shown to induce apoptosis by disrupting mitochondrial membrane potential and inhibiting the anti-apoptotic protein Bcl-Xl, as well as upregulating death receptors DR4 and DR5 [28]. Curcumin has also been identified to reduce glucose uptake and lactate production (the Warburg effect) by downregulating pyruvate kinase M2 (PKM2), an effect mediated by the suppression of the TOR-HIF1α pathway [29].

Xanthorrhizol also demonstrates significant anticancer potential, partly attributed to its monophenolic structure, a common feature of cytotoxic compounds [4]. Xanthorrhizol inhibits cancer progression through various mechanisms, including the dose-dependent attenuation of JNK phosphorylation in the MAPK signaling pathway [4]. Additionally, it induces apoptosis and suppresses tumor growth by modulating the PI3K/AKT and NF-kB pathways [30]. Xanthorrhizol also inhibits VEGF-induced angiogenesis by affecting the PI3K/Akt/eNOS signaling pathway and reducing NF-kB-dependent expression of cell adhesion molecules [31]. Collectively, this study underscores the therapeutic potential of both curcuminoids and xanthorrhizol in cancer treatment by demonstrating their ability to interfere with critical molecular pathways involved in cancer progression.

Furthermore, the cytotoxicity results showed that NADES extraction exhibited a more selective cytotoxic effect toward cancer cells (HeLa) compared to non-cancer cells (HaCaT). In contrast, traditional ethanol extraction demonstrated non-selective cytotoxicity, as shown by non-significant differences in cell viability reduction between HeLa and HaCaT cells upon 6.25 ppm treatment. The selective cytotoxicity of NADES may be attributed to their eco-friendly, non-toxic, and biodegradable nature. NADES also offer adjustable viscosity, low volatility, and a broad polarity range, making them versatile extraction solvents. Ethanol, on the other hand, is a known toxicant with health risks associated with excessive exposure, and ethanol-based extracts can sometimes contain toxic compounds derived from plant material [8].

Studies have shown variability in the cytocompatibility and cytotoxicity of different NADES on various cell lines. While some NADES display favorable biocompatibility, others may exert cytotoxic effects by increasing membrane porosity and inducing oxidative stress [32]. The cytotoxicity of NADES is influenced by the specific components and their molar ratios, with viscosity also potentially contributing to cytotoxic effects [33]. Hayyan et al. [34] noted that NADES toxicity varies depending on composition and concentration. However, our study indicates that the NADES formulation used here demonstrated good cytocompatibility, with minimal cytotoxic effects at lower concentrations, suggesting favorable biocompatibility of our NADES combination. Importantly, research has shown that NADES can be effectively applied in various natural product extractions, yielding positive biocompatibility results. For example, a NADES composed of choline chloride and glucose has proven effective in extracting anthocyanins from blueberry peels, enhancing their stability and making the extract suitable for food products [35]. Additionally, a mixture of choline chloride and fructose has successfully extracted phenolic compounds from grape skins, resulting in biocompatible extracts ideal for nutraceuticals and cosmetics [36]. Glycerol-based NADES have also been effective in stabilizing polyphenols derived from rosemary, ensuring their safety for pharmaceutical and cosmetic applications [37]. Furthermore, combinations such as glycerol and glucose have been utilized to extract chlorophylls and carotenoids from microalgae, preserving the activity of these pigments, which are valuable for food supplements and skincare products [38]. Overall, these examples highlight the adaptability and eco-friendly advantages of NADES in obtaining high-value plant-based bioactive compounds, showcasing their potential across various sectors.

## 4 Conclusion

**Fig. 8:**
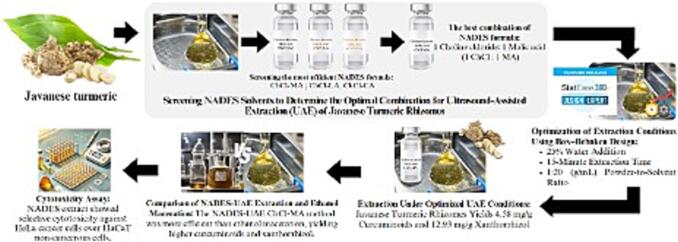
A graphical illustration demonstrating the key finding of this study: Javanese turmeric has potential as an anticancer agent using a green extraction technique. (For interpretation of the references to colour in this figure legend, the reader is referred to the web version of this article.)

This study systematically screened several salts and organic acid-based NADES combinations to identify the most effective NADES for extracting curcuminoids and xanthorrhizol from Javanese turmeric. The conditions of the selected NADES, ChCl-MA, was further refined using Box–Behnken Design, achieving yields of 4.58 mg/g for curcuminoids and 12.93 mg/g for xanthorrhizol under optimal conditions (25 % water addition, 20 mL/g solvent-to-powder ratio, and a 15-minute extraction). Additionally, the ChCl-MA NADES extract exhibited selective cytotoxicity, demonstrating greater activity against HeLa cancer cells compared to HaCaT non-cancerous cells, highlighting its potential for anticancer applications. Future research could focus on enhancing NADES formulations with optimized physicochemical properties to further improve the solubility, selectivity, and bioavailability of bioactive compounds. Investigating stability, pharmacokinetics, and applications in pharmaceuticals, cosmetics, and functional foods will ensure long-term efficacy and safety. These findings reinforce NADES combine with UAE as a viable green extraction method, paving the way for naturally derived therapeutic agents in modern bioactive compound research (Fig. 8).

## CRediT authorship contribution statement

**Donna Maretta Ariestanti:** Writing – review & editing, Writing – original draft, Visualization, Validation, Supervision, Project administration, Methodology, Funding acquisition, Conceptualization. **Abdul Mun’im:** Validation, Supervision, Resources, Project administration, Methodology, Funding acquisition, Conceptualization. **Pietradewi Hartrianti:** Writing – original draft, Visualization, Validation, Supervision, Resources, Methodology, Conceptualization. **Basmah Nadia:** Visualization, Investigation, Formal analysis, Data curation. **Erika Chriscensia:** Writing – original draft, Visualization, Investigation, Formal analysis, Data curation. **Shereen Angelina Rattu:** Writing – original draft, Visualization, Validation, Formal analysis, Data curation. **Redhalfi Fadhila:** Writing – review & editing, Writing – original draft, Visualization, Validation, Project administration, Methodology, Investigation, Data curation. **Anastacia Harianto:** Writing – review & editing, Writing – original draft, Visualization, Validation, Formal analysis. **Adelina Simamora:** Validation, Supervision, Resources, Formal analysis, Data curation. **Delly Ramadon:** Supervision, Resources, Project administration, Methodology, Investigation, Conceptualization. **Richard Johari James:** Writing – original draft, Supervision, Resources. **Fadlina Chany Saputri:** Resources, Project administration, Funding acquisition. **Mitsuyasu Kato:** Resources, Project administration, Funding acquisition. **Meidi Utami Puteri:** Writing – review & editing, Writing – original draft, Visualization, Supervision, Resources, Project administration, Funding acquisition.

## Declaration of competing interest

The authors declare that they have no known competing financial interests or personal relationships that could have appeared to influence the work reported in this paper.

## References

[1] Rahmat E., Lee J., Kang Y. (2021). Javanese turmeric (curcuma xanthorrhiza roxb.): ethnobotany, phytochemistry, biotechnology, and pharmacological activities. Evidence-Based Complement. Altern. Med..

[2] Nugraha R.V., Ridwansyah H., Ghozali M., Khairani A.F., Atik N. (2020). Traditional herbal medicine candidates as complementary treatments for COVID-19: a review of their mechanisms, pros and cons, evidence-based complement. Altern. Med..

[3] Jeliński T., Przybyłek M., Cysewski P. (2019). Natural deep eutectic solvents as agents for improving solubility, stability and delivery of curcumin. Pharm. Res..

[4] Oon S.F., Nallappan M., Tee T.T., Shohaimi S., Kassim N.K., Sa’ariwijaya M.S.F., Cheah Y.H. (2015). Xanthorrhizol: A review of its pharmacological activities and anticancer properties. Cancer Cell Int..

[5] Nurcholis W., Marliani N., Asyhar R., Minarni M. (2023). Optimized solvents for the maceration of phenolic antioxidants from curcuma xanthorrhiza rhizome using a simplex centroid design. J. Pharm. Bioallied Sci..

[6] Espino M., de los Ángeles Fernández M., Gomez F.J.V., Silva M.F. (2016). Natural designer solvents for greening analytical chemistry. TrAC - Trends Anal. Chem..

[7] Ahmad I., Arifianti A.E., Sakti A.S., Saputri F.C., Mun’im A. (2020). Simultaneous natural deep eutectic solvent-based ultrasonic-assisted extraction of bioactive compounds of cinnamon bark and sappan wood as a dipeptidyl peptidase IV inhibitor. Molecules.

[8] Hikmawanti N.P.E., Ramadon D., Jantan I., Mun’im A. (2021). Natural deep eutectic solvents (Nades): Phytochemical extraction performance enhancer for pharmaceutical and nutraceutical product development. Plants.

[9] Patil S.S., Pathak A., Rathod V.K. (2021). Optimization and kinetic study of ultrasound assisted deep eutectic solvent based extraction: a greener route for extraction of curcuminoids from Curcuma longa. Ultrason. Sonochem..

[10] Chemat F., Rombaut N., Fabiano-Tixier A.S., Pierson J.T., Bily A. (2014). Green extraction: from concepts to research, education, and economical opportunities. Green Extr. Nat. Prod. Theory Pract.,.

[11] Rachmaniah O., Muhsin M.R., Putra A.W., Rachimoellah M. (2021). Purification of curcuminoids from natural deep eutectic solvents (Nades) matrices using chromatography-based separation methods. Indones. J. Chem..

[12] Vitanti T.A.P., Kawiji K., Nurhartadi E. (2017). Effect of extraction method on Curcuma xanthorrhiza oleoresin using solar dryer to concentration of curcuminoid, total phenol and antioxidant activty. Biofarmasi J. Nat. Prod. Biochem..

[13] Ministry of Health of the Republic of Indonesia, Indonesian Herbal Pharmacopoeia II, Jakarta, 2017.

[14] Nurmiah S., Syarief R., Sukarno S., Peranginangin R., Nurmata B. (2013). Application of response surface methodology in optimizing process conditions of alkali treated cottonii (ATC). J. Pascapanen Dan Bioteknol. Kelaut. Dan Perikan..

[15] Cao J., Cao J., Wang H., Chen L., Cao F., Su E. (2020). Solubility improvement of phytochemicals using (natural) deep eutectic solvents and their bioactivity evaluation. J. Mol. Liq..

[16] Espino M., de los Ángeles Fernández M., Gomez F.J.V., Silva M.F. (2014). Natural deep eutectic solvents: the role of viscosity in extraction performance. *Sep. Purif. Technol.*.

[17] Dai Y., Witkamp G.J., Verpoorte R. (2015). The role of deep eutectic solvents in enhancing bioactive compound extraction. *Phytochem. Anal*.

[18] Dai Y., van Spronsen J., Witkamp G.J., Verpoorte R., Choi Y.H. (2013). Natural deep eutectic solvents as new potential media for green technology. *Green Chem.*.

[19] Smith P.L., Buford J.P., Yu J. (2018). Molecular transport in high-viscosity green solvents: A computational and experimental study. *J. Phys. Chem. B*.

[20] Chen Y., Wang X., Zhao L., Li F., Zhang B. (2019). Effect of solvent viscosity on mass transfer efficiency in bioactive compound extraction. *J. Green Chem.*.

[21] Zhang X., Liu X., Wu J. (2020). Improving extraction efficiency of polyphenols by adjusting NADES viscosity. *Int. J. Mol. Sci.*.

[22] Yusuf M., Yusof F., Rahman M.M., Sulaiman N. (2021). Water-assisted deep eutectic solvents for sustainable extraction of plant-based bioactives. *ACS Sustain. Chem. Eng.*.

[23] A. Mun’im, I. Ahmad, Application of Green Extraction Techniques in Herbal Medicine Development, Deepublish, Jakarta, 2021.

[24] Dai Y., van Spronsen J., Witkamp G.J., Verpoorte R., Choi Y.H. (2013). Natural deep eutectic solvents as new potential media for green technology. Anal. Chim. Acta.

[25] Hammond O.S., Bowron D.T., Edler K.J. (2017). The effect of water upon deep eutectic solvent nanostructure: an unusual transition from ionic mixture to aqueous solution. Angew. Chemie - Int. Ed..

[26] Xu M., Ran L., Chen N., Fan X., Ren D., Yi L. (2019). Polarity-dependent extraction of flavonoids from citrus peel waste using a tailor-made deep eutectic solvent. Food Chem..

[27] Nn A. (2015). A review on the extraction methods use in medicinal plants, principle, strength and limitation. Med. Aromat. Plants.

[28] Hsu K.Y., Ho C.T., Pan M.H. (2023). The therapeutic potential of curcumin and its related substances in turmeric: from raw material selection to application strategies. J. Food Drug Anal..

[29] Tomeh M.A., Hadianamrei R., Zhao X. (2019). A review of curcumin and its derivatives as anticancer agents. Int. J. Mol. Sci..

[30] Cai Y., Sheng Z., Wang J. (2022). Xanthorrhizol inhibits non-small cell carcinoma (A549) cell growth and promotes apoptosis through modulation of PI3K/AKT and NF-κB signaling pathway. Environ. Toxicol..

[31] Lee S.K., Kim M.J., Son S.H., Kim K.R., Park K.K., Chung W.Y. (2021). Xanthorrhizol suppresses vascular endothelial growth factor-induced angiogenesis by modulating Akt/eNOS signaling and the NF-κ B-dependent expression of cell adhesion molecules. Am. J. Chin. Med..

[32] Mbous Y.P., Hayyan M., Wong W.F., Looi C.Y., Hashim M.A. (2017). Unraveling the cytotoxicity and metabolic pathways of binary natural deep eutectic solvent systems. Sci. Rep..

[33] Mišan A., Nađpal J., Stupar A., Pojić M., Mandić A., Verpoorte R., Choi Y.H. (2019). The perspectives of natural deep eutectic solvents in agri-food sector. Crit. Rev. Food Sci. Nutr..

[34] Hayyan M., Mbous Y.P., Looi C.Y., Wong W.F., Hayyan A., Salleh Z., Mohd-Ali O. (2016). Natural deep eutectic solvents: cytotoxic profile. Springerplus.

[35] Grillo G., Gunjević V., Radošević K., Redovniković I.R., Cravotto G. (2020). Deep eutectic solvents and nonconventional technologies for blueberry-peel extraction: Kinetics, anthocyanin stability, and antiproliferative activity. Antioxidants.

[36] Machado T. de O.X., Portugal I., Kodel H. de A.C., Fathi A., Fathi F., Oliveira M.B.P.P., Dariva C., Souto E.B. (2024). Pressurized liquid extraction as an innovative high-yield greener technique for phenolic compounds recovery from grape pomace. Sustain. Chem. Pharm..

[37] Vieira C., Rebocho S., Craveiro R., Paiva A., Duarte A.R.C. (2022). Selective extraction and stabilization of bioactive compounds from rosemary leaves using a biphasic NADES. Front. Chem..

[38] Martins R., Mouro C., Pontes R., Nunes J., Gouveia I. (2023). Ultrasound-assisted extraction of bioactive pigments from Spirulina platensis in natural deep eutectic solvents. Bioresour. Bioprocess..

