# Supplementary material for: Ultrasonic-assisted extraction (UAE) of Javanese turmeric rhizomes using natural deep eutectic solvents (NADES): Screening, optimization, and in vitro cytotoxicity evaluation
Source: Ultrason Sonochem. 2025 Feb 12;114:107271. doi: 10.1016/j.ultsonch.2025.107271 (PMC11872070; doi:10.1016/j.ultsonch.2025.107271)
Supplement: Supplementary Data 1 [file mmc1.docx]

| Source | Sum of Squares | df | Mean Square | F-value | *p-*value |  |
| --- | --- | --- | --- | --- | --- | --- |
| **Model** | 122.34 | 9 | 13.59 | 30.86 | < 0.0001 | Significant |
| A - Water addition | 3.90 | 1 | 3.90 | 8.86 | 0.0206 |  |
| B - Extraction time | 0.0276 | 1 | 0.0276 | 0.0627 | 0.8095 |  |
| C - Powder to solvent rasio | 94.66 | 1 | 94.66 | 214.87 | < 0.0001 |  |
| Interaction |  |  |  |  |  |  |
| AB | 0.0064 | 1 | 0.0064 | 0.0145 | 0.9075 |  |
| AC | 0.2657 | 1 | 0.2657 | 0.6032 | 0.4628 |  |
| BC | 0.2862 | 1 | 0.2862 | 0.6497 | 0.4467 |  |
| A^2^ | 0.9899 | 1 | 0.9899 | 2.25 | 0.1776 |  |
| B^2^ | 6.579 | 1 | 6.579 | 1.493 | 0.9997 |  |
| C^2^ | 21.55 | 1 | 21.55 | 48.92 | 0.0002 |  |
| Residual | 3.08 | 7 | 0.4404 |  |  |  |
| Lack of Fit | 1.13 | 3 | 0.3754 | 0.7669 | 0.5692 | Non significant |
| Pure error | 1.96 | 4 | 0.4894 |  |  |  |
| Cor Total | 125.43 | 16 |  |  |  |  |
| Std. Dev | 0.6536 |  |  |  |  |  |
| R^2^ | 0.9756 |  |  |  |  |  |
| Adjusted R^2^ | 0.9443 |  |  |  |  |  |
| Predicted R^2^ | 0.8865 |  |  |  |  |  |
| Adeq Precision | 16.3870 |  |  |  |  |  |
| Mean | 9.85 |  |  |  |  |  |
| C.V % | 6.64 |  |  |  |  |  |

Supplementary Table S1 Analysis of Variance (ANOVA), factors, and their interaction factors for

Xanthorrizol prediction model.

| Source | Sum of Squares | df | Mean Square | F-value | *p-*value |  |
| --- | --- | --- | --- | --- | --- | --- |
| **Model** | 9.90 | 9 | 1.10 | 12.51 | 0.0015 | Significant |
| A - Water addition | 0.73000 | 1 | 0.73000 | 8.30 | 0.0236 |  |
| B - Extraction time | 0.0006 | 1 | 0.0006 | 0.00073 | 0.9344 |  |
| C - Powder to solvent rasio | 5.77 | 1 | 5.77 | 65.55 | <0.0001 |  |
| Interaction |  |  |  |  |  |  |
| AB | 0.0528 | 1 | 0.0528 | 0.6004 | 0.4638 |  |
| AC | 0.0916 | 1 | 0.0916 | 1.04 | 0.3413 |  |
| BC | 0.1454 | 1 | 0.1454 | 1.65 | 0.2392 |  |
| A^2^ | 1.33 | 1 | 1.33 | 15.10 | 0.0060 |  |
| B^2^ | 0.0410 | 1 | 0.0410 | 0.4663 | 0.0037 |  |
| C^2^ | 1.61 | 1 | 1.61 | 18.30 | 0.0037 |  |
| Residual | 0.6154 | 7 | 0.0879 |  |  |  |
| Lack of Fit | 0.3502 | 3 | 0.1167 | 1.76 | 0.2934 | Not significant |
| Pure error | 0.2653 | 4 | 0.0663 |  |  |  |
| Cor Total | 10.51 | 16 |  |  |  |  |
| Std. Dev | 0.2965 |  |  |  |  |  |
| R^2^ | 0.9415 |  |  |  |  |  |
| Adjusted R^2^ | 0.8662 |  |  |  |  |  |
| Predicted R^2^ | 0.4277 |  |  |  |  |  |
| Adeq Precision | 13.2459 |  |  |  |  |  |
| Mean | 3.80 |  |  |  |  |  |
| C.V % | 7.79 |  |  |  |  |  |

Supplementary Table S2 Analysis of Variance (ANOVA), factors, and their interaction factors for

Curcuminoid prediction model.

Supplementary Table S3. Table of Abbreviations

| **Abbreviation** | **Definition** |
| --- | --- |
| ANOVA | Analysis of Variance |
| BBD | Box–Behnken Design |
| BDMC | Bisdemethoxycurcumin |
| CA | Citric Acid |
| ChCl | Choline Chloride |
| CUR | Curcumin |
| DMC | Demethoxycurcumin |
| DMEM | Dulbecco’s Modified Eagle’s Medium |
| FBS | Fetal Bovine Serum |
| JNK | c-Jun N-terminal Kinase |
| LA | Lactic Acid |
| LOD | Limit of Detection |
| LOQ | Limit of Quantification |
| MA | Malic Acid |
| MAPK | Mitogen-Activated Protein Kinase |
| NADES | Natural Deep Eutectic Solvents |
| NF-kB | Nuclear Factor Kappa-Light-Chain-Enhancer of Activated B Cells |
| PI3K | Phosphoinositide 3-Kinase |
| AKT | Protein Kinase B (Akt) |
| eNOS | Endothelial Nitric Oxide Synthase |
| PKM2 | Pyruvate Kinase M2 |
| RSM | Response Surface Methodology |
| Sp-1 | Specificity Protein 1 |
| STAT3 | Signal Transducer and Activator of Transcription 3 |
| TLC | Thin Layer Chromatography |
| TOR-HIF1α | Target of Rapamycin - Hypoxia-Inducible Factor 1 Alpha |
| UAE | Ultrasound-Assisted Extraction |
| VEGF | Vascular Endothelial Growth Factor |
